# The Developmental Trajectory of Self-Injurious Behaviours in Individuals with Prader Willi Syndrome, Autism Spectrum Disorder and Intellectual Disability

**DOI:** 10.3390/diseases4010009

**Published:** 2016-02-06

**Authors:** Lauren J. Rice, Kylie M. Gray, Patricia Howlin, John Taffe, Bruce J. Tonge, Stewart L. Einfeld

**Affiliations:** 1 Brain and Mind Research Centre, University of Sydney, 94 Mallett St, Camperdown NSW 2050, Australia; lauren.rice@sydney.edu.au (L.J.R.); patricia.howlin@sydney.edu.au (P.H.); 2 Centre for Disability Research and Policy, University of Sydney, 94 Mallett St, Camperdown NSW 2050, Australia; 3 Centre for Developmental Psychiatry and Psychology, Department of Psychiatry, School of Clinical Sciences, Monash University, 270 Ferntree Gully Rd, Notting Hill VIC 3168, Australia; kylie.gray@monash.edu (K.M.G.); john.taffe@monash.edu (J.T.); bruce.tonge@monash.edu (B.J.T.)

**Keywords:** Prader Willi syndrome, autism spectrum disorder, self injurious behaviour, skin-picking, developmental trajectory

## Abstract

In the present study we examined the nature and developmental trajectory of self-injurious behaviour in Prader Willi syndrome (PWS) and autism spectrum disorder (ASD). The development of interventions is greatly aided by understanding gene to behaviour pathways, and this requires an accurate description of the behaviour phenotype, that is, which types and natural history of self-injurious behaviour are more common in PWS and ASD and which are shared with other forms of developmental disability. Self-injury displayed by individuals with PWS and individuals with ASD was compared with that reported in a group of individuals with intellectual disability due to mixed aetiology (ID group). Three self-injurious behaviours (head banging, skin-picking and hitting and/or biting self) were measured on five occasions over 18 years using the Developmental Behaviour Checklist (DBC) a well-validated caregiver report measure. Rates of skin picking were higher in individuals with PWS and hitting and/or biting self was higher in individuals with ASD compared to the ID group. Rates of head banging were similar across the three groups. Over time, skin-picking and head banging increased with age for individuals with ASD and hitting and/or biting self increased for the PWS group. In the PWS and mixed ID groups head banging decreased with age. These findings suggest that the typology and developmental trajectories of self-injurious behaviours differ between those with PWS and ASD.

## 1. Introduction

Prader Willi syndrome (PWS) is caused by the failure of the imprinted genes in the paternally derived PWS segment of chromosome 15 q11–q13 [1,2]. Cytogenetic abnormalities within this region have also been found in a small percentage of individuals with autism spectrum disorder (ASD) [3]. This has led to some interest in exploring whether PWS and ASD share phenotypic similarities. A review conducted by Dykens and colleagues (2011) found eleven behaviours that are commonly reported in both PWS and ASD; nine behaviours that occur in one disorder but not the other, and an additional ten behaviours that required further investigation [4]. The authors noted that although rates of self-injury are high in both disorders, there has not been a study that has directly compared the nature of self injury in PWS and ASD.

Up to 89% of individuals with PWS will display self-injury [5,6]; with the most common forms being skin-picking (82%), followed by nose-picking (28%), hand-biting (17%), head-banging (14%), hair-pulling (9%) and rectal-picking (6%) [7]. Cross-sectional studies have shown that skin-picking increases from early childhood to adolescence [8,9] and is also present throughout adulthood [10]. At present, there are no longitudinal studies that have investigated the developmental trajectory of skin-picking in this condition.

Self-injury also occurs in young people with autism, especially those with co-morbid intellectual disability [11]. However, only a small number of studies have specifically investigated profiles of self-injurious behaviours in ASD. Two such studies suggest that the most common forms of self-injury shown by individuals with ASD are hitting (41%) and self-biting (39%) [12,13]. Other behaviours reported in case studies include: head banging, skin-picking, and eye pressing or gouging [14,15]. Longitudinal and cross-sectional studies of ASD have found self-injurious behaviours typically decrease with age [16,17,18]. However, these studies have tended to assess self-injury generally, rather than specific types of self injury. We were unable to find studies that investigated how specific self-injurious behaviours change over the course of development in individuals with ASD.

Understanding the type of self-injury common to a disorder allows clinicians and parents to be more aware of these behaviours, increasing the likelihood of early detection and intervention. The development of interventions is greatly aided by understanding gene to behaviour pathways, and this requires an accurate description of behaviour phenotype. Understanding of the gene-to-behaviour phenotype is more feasible in PWS than in ASD given the genetic deficits are circumscribed in PWS, whereas in ASD are very heterogeneous. The comparison of self injurious behaviours in PWS and ASD contribute to understanding the specificity of the behavioural phenotype of these conditions. Similarly, understanding how these behaviours evolve over the course of development is important for development of and the assessment of more effective and appropriate interventions. Therefore, the aim of the present study is to compare the nature and developmental trajectories of self-injury in individuals with PWS and those with ASD compared with a group of individuals with an intellectual disability due to mixed causes. Self injury has consistently been shown to be associated with level of intellectual disability [11], thus, by comparing individuals with PWS or ASD to those with ID due to mixed causes we can elucidate which behaviours are specific to each syndrome and which are better accounted for by co-existing intellectual disability.

Based on the research summarised above, we hypothesise that:

1. Types of self-injurious behaviour will differ between the two syndrome groups, with skin-picking being most commonly displayed by individuals with PWS and hitting and biting most commonly displayed by individuals with ASD.
2. All three forms of self-injury will decrease with age in individuals with ASD.
3. Skin-picking will remain stable with age in individuals with PWS.

## 2. Results

Means and standard deviations for each of the three types of self- injury at the five time points are summarized in Table 1. Results from the regression analysis are summarized in Table 2.

**Table 1:** Rates of behaviours over time.

	Bangs Head			Hits and/or Bites Self			Picks Skin		
	ASD	PWS	mixed ID	ASD	PWS	mixed ID	ASD	PWS	mixed ID
Time 1	0.12 (98)	0.13 (39)	0.11 (384)	0.34 (98)	0.22 (40)	0.23 (386)	0.33 (386)	0.90 (.40)	0.27 (386)
Time 2	0.19 (78)	0.12 (26)	0.10 (307)	0.37 (78)	0.31 (26)	0.20 (306)	0.33 (306)	0.88 (26)	0.30 (306)
Time 3	0.19 (83)	0.23 (26)	0.07 (292)	0.33 (83)	0.27 (26)	0.18 (292)	0.35 (292)	0.92 (.26)	0.32 (292)
Time 4	0.17 (81)	0.24 (29)	0.09 (290)	0.38 (81)	0.41 (.29)	0.19 (290)	0.28 (289)	0.79 (29)	0.28 (289)
Time 5	0.19 (72)	0.04 (25)	0.06 (272)	0.32 (72)	0.52 (25)	0.18 (272)	0.31 (271)	0.88 (25)	0.31 (271)

Proportions (prevalence) for the three self-injurious behaviours (banging head, hitting and/or biting self) for three syndrome groups (ASD, PWS and mixed ID group) across the five time points.

**Table 2:** Random effects logistic regressions.

		Bangs Head	Hits and/or Bites Self	Picks Skin
Age		**0.94**	*0.97*	1.01
Devel. dis group (ref: mixed ID)	ASD	0.61	*3.27*	0.95
	PWS	1.54	0.32	***211.43***
Interaction of age and devel. dis group	Age × ASD	**1.11**	1.02	**1.08**
	Age × PWS	1.07	**1.16**	0.99
level of ID (ref: mild)	moderate	***4.76***	***4.28***	1.06
female		0.70	1.04	**2.24**
intercept		**0.02**	**0.05**	***0.11***
Number of observations		2102	2104	2103
Number of participants		524	524	524

Random effects logistic regressions of three DBC items on age, etiology of ID, age by etiology interaction, level of ID and gender (odds ratios). Typeface code for *p*-values: >0.05, <0.05, <0.01, <0.001.

Results are given below for each of the 3 items.

### 2.1. Bangs Head

The odds of engaging in this behaviour declined with age in the group with mixed cause of ID. A similar pattern occurred in the Prader Willi group, but not in the ASD group, whose odds of engaging in head banging increased with age. After age effects were accounted for there were no overall average differences between the groups in the odds of engaging in this behaviour. Those with moderate ID had greater odds of engaging in head banging than those with mild ID, on average across groups, age and gender. Gender was not associated with the propensity to engage in this behaviour.

### 2.2. Hits and/or Bites Self

The odds of engaging in these behaviours increased with age among those with PWS, but not among those in the mixed ID and ASD groups. After accounting for age effects those with ASD had higher odds on average than those in the comparison group, but those with PWS did not. After accounting for age and group effects the odds of engaging in these behaviours were higher on average among those with moderate ID than among those with mild ID, and not associated with gender.

### 2.3. Scratching and/or Picking Skin

The odds of engaging in these behaviours increased with age among those with ASD, but not among those in the comparison and PWS groups. After accounting for age effects those with PWS had much higher odds on average than those in the comparison group, but those with ASD did not have different odds from those in the mixed ID group. After accounting for age and group effects the odds of engaging in these behaviours were higher on average among females than among males, and not associated with level of ID.

## 3. Discussion

The aim of this study was to investigate the nature and developmental trajectory of self-injurious behaviours in individuals with PWS and individuals with ASD compared to a group of individuals with intellectual disability of mixed causes. In line with our first hypothesis, we found that hitting and/or biting self was more common in ASD and skin-picking more common in PWS than in the mixed ID group; rates of head banging were similar across the three groups*.* In contrast with our second hypothesis, however, both head banging and skin picking increased with age in the ASD group. In the PWS group skin picking remained stable with age, as expected; although hitting and/or biting self increased. Head banging decreased in both the PWS and mixed ID group.

Our findings confirm the inclusion of skin-picking as part of the behaviour phenotype of PWS, but not that of ASD and the ID group. As predicted, we found rates of skin-picking remained fairly stable with age for individuals with PWS. This is in line with previous research conducted in adults with PWS [10]. However, it is inconsistent with cross-sectional studies that suggest skin-picking increases from early childhood to late adolescence [8,9]. In the current, longitudinal study, the mean age of PWS participants increased from 15 years when first seen to 30 years at Time 5. Therefore, it is likely that inconsistencies in study findings are due to differences in the developmental periods examined.

Skin picking did not decrease with age in individuals with PWS, suggesting that this is a stable feature of PWS and is probably directly associated with the genomic lesion. At present the physiological mechanism of skin picking is unknown and evidence for effective behavioural and pharmaceutical treatments is limited [19]. Understanding the causal mechanism of skin picking will assist the development of targeted interventions.

It is interesting to note that hitting and/or biting self increased only in the PWS group. The reasons remain to be understood. Temper outbursts are characteristic of PWS and are usually described as poorly directed forms of aggression. It may be that hitting and/or biting self is another form of poorly directed aggression.

We found a paucity of data on specific types of self injury in ASD. In this study we found hitting and/or biting self was more common in those with ASD than the mixed ID group but head banging and skin picking was not. This is consistent with previous studies that have also reported hitting and biting self to be among the most common forms of self-injury in individuals with ASD [12,13,20,21].

In the ASD group, we found hitting and/or biting self remained stable while skin-picking and head banging increased over time. Other studies have noted a decrease in self-injurious behaviours in individuals with ASD [17,18]. However, these other studies did not examine the specific types of self injury, rather examined self injury more generally.

Gender was not associated with any of the three self injurious behaviours, suggesting that these behaviours occur equally for males and females. In relation to level of ID, we found that individuals with moderate ID are more likely to bang their head and hit and/or bit themselves than are those with mild ID. There was no difference found between the two ID groups for skin picking. The developmental trajectories described above remained the same when the analysis was restricted to only those with mild ID. Suggesting that the age-related changes are independent of level of intellectual disability.

In this study, only three forms of self-injury were examined. It is possible that other forms of self injury may provide different results. A second limitation to the present findings is that the mean age of PWS participants was higher than in the ASD group across the five time points. A third limitation is that information on specific treatments including the use of psychotropic medication was not collected and it is possible that some participants received treatment which affected behaviours. However, we do know that only 10% of participants had received specialist services. Furthermore, the evidence for an effective medication in reducing self injurious behaviours in developmental disabilities is limited (King, 2000, #47360). In recent years, topiramate and N-acetylcysteine have been shown to have some effect on skin picking in PWS [22,23,24] but it is unlikely that participants were prescribed either of these medications at the time of data collection.

## 4. Materials and Methods

### 4.1. Participants

Participants were part of the Australian Child to Adult Development Study (ACAD), which collected information from carers of a cohort of individuals with intellectual disability at five time points over 18 years (1991–2008) [25] (see Table 3 for summary of group characteristics). 

**Table 3:** Participant demographics.

	Mean Age (SD)			n			% of Females		% Individuals with Mod ID			
	ASD	PWS	mixed ID	ASD	PWS	ASD	PWS	mixed ID	ASD	ASD	PWS	mixed ID
Time 1	8.14 (4.01)	14.84 (6.12)	11.89 (4.29)	98	40	386	16	33	44	45	15	56
Time 2	12.03 (4.15)	18.69 (6.77)	16.27 (4.45)	78	26	307	18	27	43	46	15	58
Time 3	14.93 (4.26)	21.31 (6.75)	19.29 (4.49)	84	26	292	17	35	45	45	12	57
Time 4	18.43 (4.23)	25.12 (6.44)	23.15 (4.44)	81	29	290	17	28	47	46	14	57
Time 5	24.03 (4.45)	29.22 (6.54)	28.36 (4.47)	72	25	272	19	32	47	46	12	60

Mean age (standard deviation), gender, number of participants and level of intellectual disability (mild or moderate) for each participant group (autism spectrum disorder, Prader Willi syndrome and intellectual disability due to other causes (mixed ID group) at the five time points data was collected.

### 4.2. Measures

Self-injurious behaviour was measured using the Developmental Behaviour Checklist (DBC) [26,27]. The DBC is a caregiver report measure designed to assess a broad range of behavioural and emotional disturbances in people with intellectual disability. It has been used at each phase of our longitudinal follow-up study of individuals with intellectual disability and genetic disorders [26]. The DBC-P (Primary Carer Version) [26] has 96 items and was completed for those aged between 4 and 17 years. The DBC-A (Adult Version) [27] was used for participants aged 18 years and older. The DBC-P has high inter-rater reliability (ICC = 0.80), high internal consistency (0.941), high concurrent validity with other measures of behaviour disturbance, and high criterion group validity in distinguishing psychiatric cases from non-cases (*t* = 7.8, *p* < 0.001) [25,28]. Similarly, the DBC-A has high inter-rater reliability (ICC = 0.72), high internal consistency (0.95) and high concurrent validity with the Aberrant Behaviour Checklist (0.63), clinical ratings (0.52) and the Psychiatric Assessment Schedule for Adults with Developmental Disabilities Checklist (0.61) [27,29]. Validity of each item was assessed through interviews with parents and carers to determine whether their understanding of each item was the same as that of the experienced child psychiatrist [26].

The DBC asks caregivers to rate behaviours on a three-point scale, where 0 = not true as far as you know, 1 = somewhat or sometimes true and 3 = very true or often true. The DBC contains three items specifically focusing on self injury and data on these behaviours have been collected throughout the longitudinal study, allowing for comparisons over time. The three types of self-injury assessed are: (1) bangs head (ICC = 0.48), (2) hits and/or bites self (ICC = 0.66) and (3) scratches or picks his/her skin (ICC = 0.79) [26].

### 4.3. Procedure

The cause of intellectual disability was established through medical and genetic history and, where necessary, further investigations were undertaken. For the majority of participants, level of intellectual impairment (mild, moderate, severe, profound) was determined through standard IQ tests conducted by experienced psychologists [26,27]. For the remaining participants, level of intellectual disability was ascertained through clinical or educational records. There were no individuals with PWS who had profound and very few with severe intellectual disability and therefore only participants in the ASD and ID groups who had mild and moderate intellectual disability were included in this study. Data was collected over an 18-year time period. The average number of years between each of the data collection time points was 4.1 years (Times 1–2), 2.8 years (Times 2–3), 3.5 years (Times 3–4), and 5.6 years (Times 4–5).

### 4.4. Statistical Analysis

Random effects logistic regression was used to model the propensity of participants to engage in each of 3 behaviours (bangs head, bites self, picks skin) as a function of age at occasion of measurement (5 occasions), cause of ID (mixed, autism, PWS), interaction between age and cause of ID, level of ID (mild, moderate) and gender.

## 5. Conclusions

The primary aim of this study was to investigate the similarities and differences in self-injury in individuals with PWS compared to individuals with ASD. Our findings suggest that although rates of self-injury are high in both groups, the nature and developmental trajectories of these behaviours are significantly different.

We found that the forms of self-injury most common to PWS and ASD (skin-picking for PWS and hitting and/or biting self for ASD) do not decline with age. This suggests that effective interventions need to be found to reduce the persistence of these behaviours.

## References

[1] Dykens E.M., Roof E. (2008). Behavior in Prader-Willi syndrome: Relationship to genetic subtypes and age. J. Am. Acad. Child Adolesc. Psychiatry.

[2] Holm V.A., Cassidy S.B., Butler M.G., Hanchett J.M., Greenswag L.R., Whitman B.Y., Greenberg F. (1993). Prader-Willi syndrome: Consensus diagnostic criteria. Pediatrics.

[3] Liu X., Takumi T. (2014). Genomic and genetic aspects of autism spectrum disorder. Biochem. Biophys. Res. Commun..

[4] Dykens E., Lee E., Roof E. (2011). Prader-Willi syndrome and autism spectrum disorders: An evolving story. J. Neurodev. Disord..

[5] Einfeld S., Smith A., Durvasula S., Florio T., Tonge B. (1999). Behavior and emotional disturbance in Prader-Willi syndrome. Am. J. Med. Genet. A.

[6] Holland A.J., Whittington J.E., Butler J., Webb T., Boer H., Clarke D. (2003). Behavioural phenotypes associated with specific genetic disorders: Evidence from a population-based study of people with Prader-Willi syndrome. Psychol. Med..

[7] Symons F.J., Butler M.G., Sanders M.D., Feurer I.D., Thompson T. (1999). Self-injurious behavior and Prader-Willi Syndrome: Behavioral forms and body locations. Am. J. Ment. Retard..

[8] Wigren M., Hansen S. (2005). ADHD symptoms and insistence on sameness in Prader Willi syndrome. J. Intellect. Disabil. Res..

[9] Dimitropoulos A., Feurer I.D., Butler M.G., Thompson T. (2001). Emergence of compulsive behavior and tantrums in children with Prader-Willi syndrome. Am. J. Ment. Retard..

[10] Dykens E.M., Hodapp R.M., Walsh K., Nash L.J. (1992). Adaptive and maladaptive behavior in Prader-Willi Syndrome. J. Am. Acad. Child Adolesc. Psychiatry.

[11] McClintock K., Hall S., Oliver C. (2003). Risk markers associated with challenging behaviours in people with intellectual disabilities: A meta-analytic study. J. Intellect. Disabil. Res..

[12] Buono S., Scannella F., Palmigiano M.B. (2010). Self-injurious behavior: A comparison between Prader-Willi syndrome, Down syndrome and autism. Life Span Disabil..

[13] Richards C., Oliver C., Nelson L., Moss J. (2012). Self injurious behaviour in individuals with autism spectrum disorder and intellectual disability. J. Intellect. Disabil. Res..

[14] Brady D.O., Smouse A.D. (1978). A simultaneous comparison of three methods for language training with an autistic child: An experimental single case analysis. J. Autism Child. Schizophr..

[15] O’Reilly M., Sigafoos J., Lancioni G., Edrisinha C., Andrews A. (2005). An examination of the effects of a classroom activity schedule on levels of self-injury and engagement for a child with severe autism. J. Autism Dev. Disord..

[16] Bishop S.L., Richler J., Lord C. (2006). Association between restricted and repetitive behaviors and nonverbal IQ in children with autism spectrum disorders. Child Neuropsychol..

[17] Esbensen A.J., Seltzer M.M., Lam K., Bodfish S.L., James W. (2009). Age-related differences in restricted repetitive behaviors in autism spectrum disorders. J. Autism Dev. Disord..

[18] Shattuck P.T., Seltzer M.M., Greenberg J.S., Orsmond G.I., Bolt D., Kring S., Lounds J., Lord C. (2007). Change in autism symptoms and maladaptive behaviors in adolescents and adults with an autism spectrum disorder. J. Autism Dev. Disord..

[19] Ho A.Y., Dimitropoulos A. (2010). Clinical management of behavioral characteristics of Prader-Willi Syndrome. Neuropsychiatr. Dis. Treat..

[20] Canitano R. (2006). Self injurious behavior in autism: Clinical aspects and treatment with risperidone. J. Neural. Transm..

[21] Duerden E.G., Oatley H.K., Mak-Fan K.M., McGrath P.A., Taylor M.J., Szatmari P., Roberts S.W. (2012). Risk factors associated with self-injurious behaviors in children and adolescents with autism spectrum disorders. J. Autism Dev. Disord..

[22] Miller J.L., Angulo M. (2014). An open-label pilot study of N-acetylcysteine for skin-picking in Prader-Willi Syndrome. Am. J. Med. Genet. A..

[23] Shapira N.A., Lessiq M.C., Lewis M.H., Goodman W.K., Driscoll D.J. (2004). Effects of topiramate in adults with Prader-Willi Syndrome. Am. J. Ment. Retard..

[24] Smathers S.A., Wilson J.G., Nigro M.A. (2003). Topiramate effectiveness in Prader-Willi syndrome. Pediatr. Neurol..

[25] Einfeld S.L., Piccinin A.M., Mackinnon A., Hofer S.M., Taffe J., Gray K.M., Bontempo D.E., Hoffman L.R., Parmenter T., Tonge B. (2006). Psychopathology in young people with intellectual disability. JAMA.

[26] Einfeld S., Tonge B.J. (2002). Manual for the Developmental Behaviour Checklist.

[27] Mohr C., Tonge B.J., Einfeld S., Taffe J. (2011). Manual for the Developmental Behaviour Checklist for Adults (DBC-A).

[28] Einfeld S., Tonge B.J. (1995). The Developmental Behavior Checklist: The development and validation of an instrument to assess behavioral and emotional disturbance in children and adolescents with mental retardation. J. Autism Dev. Disord..

[29] Mohr C., Tonge B.J., Einfeld S.L. (2005). The development of a new measure for the assessment of psychopathology in adults with intellectual disability. J. Intellect. Disabil. Res..

